# Class I Biocompatible DLP-Printed Acrylate Impairs Adhesion and Proliferation of Human Mesenchymal Stromal Cells in Indirect Cytotoxicity Assay

**DOI:** 10.1155/2023/8305995

**Published:** 2023-10-14

**Authors:** Franziska Alt, Christiane Heinemann, Benjamin Kruppke

**Affiliations:** Institute of Materials Science, Faculty of Mechanical Science and Engineering, Technical University Dresden, TU Dresden, Budapester Str. 27, 01069 Dresden, Germany

## Abstract

The popular method of digital light processing 3D printing (DLP) for complex and individual laboratory equipment requires materials that are as inert as possible for use in contact with cells for subsequent investigations. However, the per se incomplete curing of acrylate resins by UV light leaves residuals that are not suitable for cell culture application. Therefore, we evaluated the cytotoxicity of four commercially available acrylate resins with bone marrow-derived human mesenchymal stromal cells (BM-hMSC) in an indirect cytotoxicity test. This involved incubating the printed cylinders in Transwell™ inserts for 7 days. While the degree of crosslinking did not increase significantly between freshly printed and stored samples (3 weeks in ambient conditions), the storage improved the material's performance in terms of cytocompatibility. The DNA amount and LDH activity showed a direct influence of the resin residuals on cell adhesion. The class I acrylate Surgical Guide™ left no adherent cells after 7 days, regardless of previous storage. In comparison, the Basic Ivory™ resin after storage allowed same amount of adherent cells after 7 days as the polystyrene reference. We conclude that resin residuals of certain materials are released, which allows the use of the resins in indirect contact with cells thereafter.

## 1. Introduction

In the research field of medical technology or dental medicine—where contact of a material with biological tissues and cells is most frequently intended—the production of noncommercial and specific components using various 3D printing processes is increasingly established as easy to use and cost-efficient in-house production options. Today, one finds many examples of complex examination setups or laboratory utensils like stirrers, bioreactors, and sample holders in many labs. There are different approaches for 3D printing, which all have their own advantages and disadvantages. When the material is melted and then deposited through a needle in the form of a strand in a defined pattern, this is known as fused deposition modeling (FDM). The resolution of the printed construct is determined by the diameter of the needle. Higher resolution can be achieved, for example, by digital light processing (DLP). In this method, liquid polymer resins are polymerized or crosslinked to a solid state by an area-to-area exposure to UV light [1]. Due to the limited penetration depth of the light into the liquid resin, the component must be printed layer by layer. For this, the sample table is immersed in the liquid resin from the above and the pattern of the respective layer to be printed is displayed on an LCD screen, through which the UV light shines and crosslinks the resin. The table then moves upwards layer by layer [2]. After the printing process, manufacturers recommend a cleaning step in an isopropanol ultrasonic bath and postcuring, which can reduce the sticky haptic of the final product [3].

The resins used in DLP are composed of a prepolymer (oligomer), diluents (or monomers), and photoinitiators, while some formulations contain further on coinitiators, light stabilizer, thermal stabilizer, colourants, plasticisers, and additives in general [4]. The majority of used UV-curable oligomers are acrylates [5], which means they consist of derivatives of acrylic or methacrylic acid [6]. Those chemical structures contain a vinyl group built by two double-bonded carbon atoms, which are directly attached to the carbonyl carbon of the ester group [7]. The crosslinking of acrylates is typically induced by ionic or, mainly, radical polymerization [8]. For the latter, the initial radicals are provided by photoinitiators, which further interact with acrylate monomers to initiate radical chain polymerization [9, 10]. However, complete crosslinking is very unlikely, since during the crosslinking process, the mean free path length of the monomers increases, while the mobility decreases [11]. It has been shown that after crosslinking, resin residuals such as monomers or photoinitiators can continue to diffuse out of the material, potentially causing harmful biological effects such as local and systemic toxicity and allergic reactions, as observed and discussed by different groups [12–17]. In the context of toxicity, both pure monomers [12–14], pure photoinitiators [16, 17], and also commercially available systems [17] were investigated and discussed. Among other things, Mondschein et al. provided an overview of three photoinitiators and discussed their effect on six cell populations, summarizing the different degrees of cytotoxicity of photoinitiators. Based on the results found, it can be concluded that the resin can be formulated appropriately for its application [16, 17]. Nevertheless, there are some acrylic resins, which have been awarded class I (e.g., Raydent Surgical Guide Resin) and class II (e.g., EnvisionTEC E-Shell 300) certificates, and it can be assumed that these are not cytotoxic within certain limits and would therefore be the first choice for indirect cell cultures. There are no international standards for biocompatibility classification; wherefore, classes could vary depending on the country of certification. Raydent Surgical Guide was certificated as class I by rule 5 of Annex IX, MDD 93/42/EEC as amended by Directive 2007/47/EC, which declares it an invasive device with respect to body orifices which are not intended for connection to an active medical device and are intended for transient use. In the same certification standard, class II would refer to materials for use in contact with the blood, cells, tissue, and organs, but not with the heart, the central circulatory system, or the central nervous system.

The motivation for this research is the planned application of DLP-printed impellers in a spinner flask for the expansion of BM-hMSC. In those expansion spinner flasks, the impeller is connected to a magnetic-activated stirrer rod, while being immersed in cell culture medium. The interval stirring distributes cells homogeneously in the medium, preventing sedimentation. Therefore, fabricated components do not come into direct contact with the cells, which is why no necessity of class II was deemed.

Based on that, the aim of this publication is to investigate the influence of the storage time of printed acrylic resins on the cell viability of bone marrow-derived human mesenchymal stromal cells (BM-hMSCs). The term “cell viability” is understood as the number of healthy cells after interaction with one sample. The hypothesis is that with longer storage time of printed resins at ambient conditions, the amount of residual monomers and photoinitiators in the printed construct will be decreased and thus lower the amount of released molecules, when stored in a (cell culture) liquid. This monomer decrease during storage may thus cause compatibility in the interaction of the printed materials with cells.

Adapted from the intended use case of cell expansion in spinner flasks, an indirect cytotoxicity test was performed over 7 days, while 10 to 14 days are usually sufficient for cell expansion; our test was stopped after 7 days because of clearly visible material-cell interactions, such as insufficient adhesion and signs of cell death. The material specimens were stored in well-plate inserts with porous membranes (3 *μ*m), which were placed in cell culture well plates containing the cells on the bottom. Therefore, only resin residuals released into the medium can interact with the cells, consequently excluding the interaction of cells with the printed specimen surfaces. Four commercial acrylic resins were investigated for their suitability for use in cell culture: Tough Clear™ (TC, Zortrax), Premium Flex™ (PF, Liqcreate), Basic Ivory™ (BI, Zortrax), and Surgical Guide™ (SG, Raydent). SG is certified class I biocompatible for transient intraoral use.

## 2. Methods

### 2.1. DLP 3D Printing

The models were created in SOLIDWORKS 2021 (Dassault Systèmes) and printed with the Zortrax Inkspire (Zortrax, Poland) with the respective recommended print settings (supplementary table 1). After printing, the specimens were cleaned in an isopropanol ultrasound bath for 3 min and postcured on both sides twice for 10 min each under UV at a wavelength of 365 nm (CL-1 Crosslinker, Herolab, Germany), as suggested of the printer's manufacturer [3]. Three samples each were transferred to the cell culture after printing and postcuring as well as after 3 weeks of storage at room temperature and at ambient conditions and were labeled “fresh” and “stored” accordingly. All samples for cell culture were autoclaved before use.

### 2.2. Determination of Crosslinking Degree by FT-IR

Fourier-transformed infrared spectroscopy (FT-IR) was performed in attenuated total reflection (ATR) mode with a resolution of 2 cm^−1^ over 16 scans and from 4000 cm^−1^ to 400 cm^−1^ (Spectrum Two, PerkinElmer, USA).

The degree of crosslinking was determined from the C=C shear vibration at 1410 cm^−1^ and the carbonyl vibration at 1720 cm^−1^. To determine boundaries of the vibration for area integration, peak analysis was used in Origin 2019 (OriginLab). The curing ratio (CR) of the liquid and crosslinked system was calculated by dividing the area content of the C=C vibration by that of the carbonyl group, which served as an internal standard [18]. The curing level (CL) is then calculated according to

(1) $$\begin{array}{c} \text{CL}=100-\left(\frac{{\text{CR}}_{\text{cured}}}{{\text{CR}}_{\text{liquid}}}*100\right). \end{array}$$

With this calculation, CL is ranging between 0% (not crosslinked, liquid system) and 100% (completely crosslinked, all C=C groups were polymerized).

### 2.3. Cell Culture

BM-hMSCs (Caucasian male, 25 years, obtained from Medical Clinic I, University Hospital Dresden; ethical approval EK 367072019) were expanded in Dulbecco's modified Eagle's medium (DMEM) low glucose (+1% P/S, +20 mM L-glutamine, and +10% FBS) in a humidified atmosphere at 37°C and 7% CO_2_ and used for experiments in passage 5.

In a 24-well plate (TPP, Switzerland), 12 000 BM-hMSCs were seeded per well and expanded for 3 days. Subsequently, the medium was changed, and the cytotoxic experiment was started by mounting the specimens in commercially available well-plate inserts (PET membrane, 3 *μ*m pore size, Sarstedt, Germany). The time of indirect cultivation refers to the time intervals after material application of 24 hours, 3 days, and 7 days, respectively. At these time points, cell number was determined spectrophotometric using LDH and DNA in the supernatants. Cell count analysis and microscopy of adhered cells were performed after 24 hours and 7 days. Wells without material exposure served as reference in each case. All experiments were performed in triplicates. First, we summarize and explain in detail below how the cell numbers of living and dead cells and the number of adherent and nonadherent living cells were determined.

### 2.4. Living Adherent Cells

Living adherent cells were determined on the plate from LDH after freezing.

### 2.5. Dead Cells

Since dead cells are not adherent, their amount was calculated from LDH of not-frozen cultivation medium (supernatant).

### 2.6. Nonadherent Living Cells

Nonadherent cells are located in the cultivation medium; wherefore, the supernatant was analyzed. While LDH of dead cells is already released into the medium, LDH of living cells is needed to be released by cell membrane destruction by freezing the medium.

It is worth to notice that subzero temperatures denature LDH, but in an unknown efficiency [19], why the number of nonadherent living cells cannot be reliably determined by LDH. Meanwhile, DNA is stable during freezing, giving the amount of nonadherent living and dead cells. With DNA (frozen) equals dead and living cells in the medium and LDH (not frozen) equals dead cells in the medium, both methods can be combined to calculate the amount of nonadherent but still living cells (NALcells) as follows:

(2) $$\begin{array}{c} {\text{DNA}}_{\text{frozen}}-{\text{LDH}}_{\text{notfrozen}}=\text{NALcells}. \end{array}$$

### 2.7. DNA

DNA was measured using Quant-iT™ PicoGreen™ dsDNA Reagent (Thermo Fisher Scientific, USA) in black microtiter plates (Nunc, Denmark) according to the manufacturer's instructions. Plates with adherent cells were frozen at -80°C without the addition of liquid and then lysed with 1% Triton in PBS. Furthermore, liquid samples of cell culture medium were frozen at -80°C to measure DNA of all nonadherent (dead and living) cells after cultivation. Liquid samples were not treated with Triton in PBS.

In each well in a 96er well plate, 10 *μ*L of sample lysate is added followed by 200 *μ*L of PicoGreen working solution (1 : 400 diluted PicoGreen in 200 mM Tris base and 20 mM EDTA in ddH2O with pH 7.5 adjusted with 4 M HCl). After light-protected incubation for 10 min at RT and shaking, fluorescence is measured at 485 nm excitation and 520 nm emission using a plate reader (Infinite M200Pro, Tecan, Switzerland). The DNA amount was correlated with the number of cell nuclei using calibration lines of cell lysates of pure BM-hMSCs with defined cell numbers. Samples were analyzed in duplicates.

### 2.8. LDH

LDH activity was determined from the total activity of LDH in the cell lysates, as well as supernatants using the LDH cytotoxicity detection kit (Takara, Saint-Germain-en-Laye, France). Plates with adherent cells were frozen at -80°C without the addition of liquid and then lysed with 1% Triton in PBS. For the LDH activity measurements in the supernatant, the culture medium was measured not frozen, without treatment of Triton in PBS. Thus, the number of dead cells in the supernatant (LDH was already released from the cells without freezing) could be concluded.

For each sample, 50 *μ*L lysate is mixed with 50 *μ*L of LDH substrate buffer in a 96er well plate and incubated at RT for 15 min with constant shaking, protected from light. The reaction is stopped with 50 *μ*L each of 0.5 M HCL, and the absorbance is determined using a plate reader (Infinite M200Pro, Tecan, Switzerland) at 492 nm. The LDH amount was correlated with the cell number using calibration lines of cell lysates of pure BM-hMSC with defined cell numbers. Samples were analyzed in duplicates.

### 2.9. Release Test

Specimens of 10 mm in diameter and 10 mm in height were stored in a descending specimen to DMEM ratio and stored at 37°C for 7 days. For this, 2 specimens were immersed in 1 mL DMEM (“2 in 1 mL”), 1 specimen in 1 mL (“1 in 1 mL”), and 1 specimen in 2 mL DMEM (“1 in 2 mL”). Medium without indirect material cultivation was equally incubated as reference. Medium change was performed in cell culture rhythm after 3 to 4 days. Afterwards, each specimen was analyzed by FT-IR in ATR mode with a resolution of 2 cm^−1^ over 8 scans and from 4000 cm^−1^ to 400 cm^−1^ (Spectrum Two, PerkinElmer, USA) to determine a wash-out effect of residuals as a function of the chemical structure of the resin bulk material after incubation. A baseline correction as performed by Spectrum Software (PerkinElmer, USA). Spectrums were averaged and normalized from 0 to 100. The tests were performed in triplicate.

### 2.10. Statistics

Data bars show means from triplicates. The standard deviation is shown as error bars. Student's *t*-test with two independent samples was performed between selected and highlighted results with a confidence level of 0.05. Existing and nonexisting significances are marked accordingly as “^∗^” and “n. s.” in the graph.

## 3. Results and Discussion

**Figure 1:**
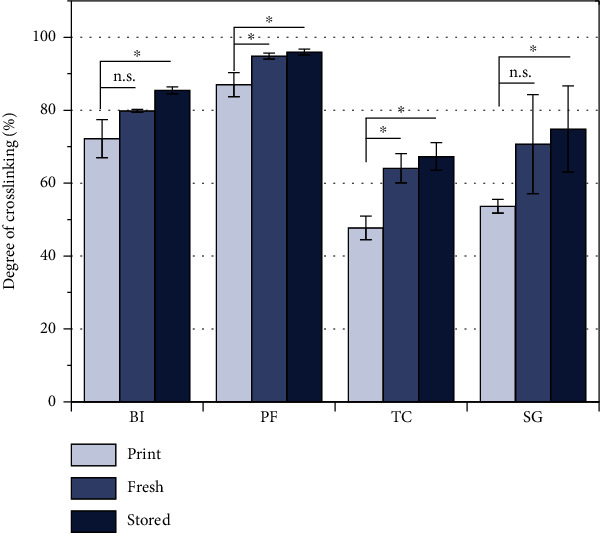
Curing level of acrylic resins directly after printing (“print”), after postcuring (“fresh”) under UV light (365 nm, 2× 10 min), and after 3 weeks of storage at ambient conditions (“stored”), determined by ATR FT-IR. Data plotted as means (*n* = 3) with SD as error bars. Significances (*p* < 0.05) are indicated with “^∗^.”

The specimen was prepared by DLP, as suggested by the manufacturer and described in the methodology. The degree of crosslinking of the resins was determined by FT-IR directly after printing (“print”), after postcuring (“fresh”), and after storage at ambient conditions for 3 weeks (“stored”). The curing level of all resins was increased by postcuring (Figure 1, “print” vs. “fresh”), which inevitably reduces the proportion of uncrosslinked monomers. Storage for 3 weeks increases the curing level to a minor degree without being significant (“fresh” vs. “stored” in Figure 1). This slight increase of curing level is related to the decreased mean free path length of the monomers, so further crosslinking is hardly possible. It can be concluded that the radical crosslinking cascade is ongoing during storage, increasing the curing level and therefore decreasing the amount of resin residuals. A low proportion of uncrosslinked monomers, which could potentially diffuse from the crosslinked volume, is instrumental in lowering cytotoxicity [20, 21]. However, the free resin residuals may undergo crosslinking reactions or diffuse out of the bulk material over time. As a result, their proportion can be reduced by prolonged storage or washing processes to improve cell compatibility [15, 20].

**Figure 2:**
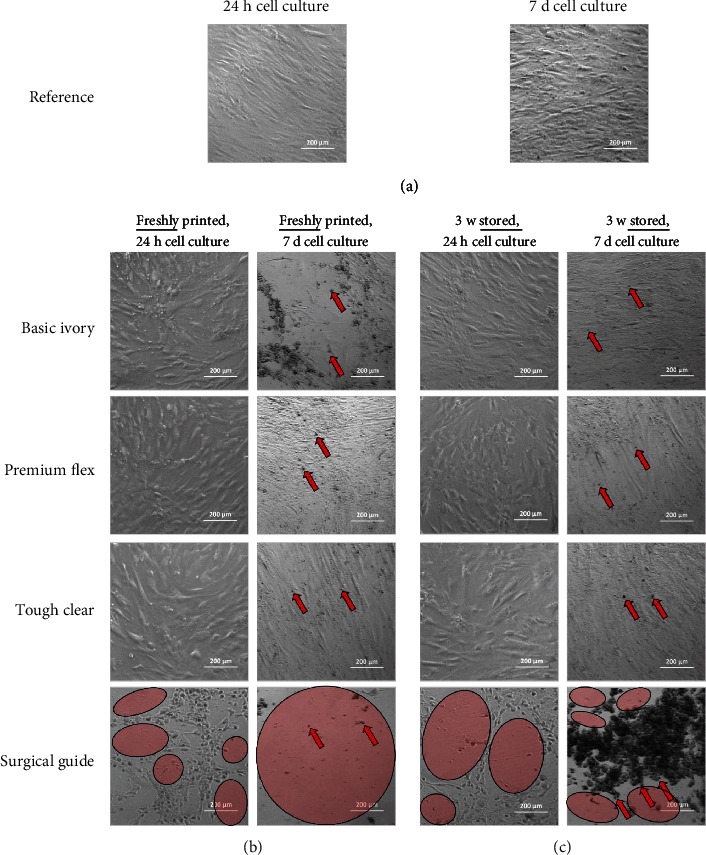
Cell morphology after indirect cultivation to crosslinked acrylate resins in fresh and stored condition, after 24 hours and 7 days of indirect cell cultivation. Signs of apoptosis are indicated with red arrows. Areas without cells due to detachment are highlighted with red circles.

Based on the knowledge that the reaction of the resins is not complete or is still in progress, the indirect interaction with cells through the storage medium is of interest for many research applications. In the indirect cell experiments, BM-hMSCs first adhered to the polystyrene (PS) well plate before the materials were transferred into the well inserts and thus into the medium and indirect cell contact. Cells that were not exposed to any material were used as reference. The microscopy images provide an overview of the changes in cell morphology caused by indirect cultivation to the material after 24 hours and 7 days (Figure 2). Focusing on the BM-hMSCs of the reference (Figure 2(a)), they started with about 50% confluence and showed the typical slenderly elongated and aligned morphology, even after 7 days of cultivation, during which time approx. 80% confluence was reached.

The influence of the various fresh materials is depicted in Figure 2(b). BI-fresh, PF-fresh, and TC-fresh show the same elongated morphology compared to the reference, while SG-fresh showed a drastic impact on cell morphology and adhesion after 24 h indirect cultivation. The cells lose adherence to the plate but continue to clump together. After 7 days of indirect cultivation to the fresh material, these morphological changes are even more pronounced. Large area detachment and clumping of cells can be seen after indirect cultivation of 7 days to BI-fresh and SG-fresh; no BM-hMSC typical morphology is present. In contrast, cells were still adherent after indirect cultivation to PF-fresh and TC-fresh and could also visibly proliferate, as confluence of approx. 80% was reached. Nevertheless, darker spots (marked with red arrows) indicate apoptosis for PF-fresh and TC-fresh, as well.

For the stored materials BI-stored, PF-stored, and TC-stored (Figure 2(c)), the cell morphology after 24 hours of indirect culture is comparable to the reference, whereas SG-stored induces cell detachment, just as the SG-fresh did. The impact of those stored materials after 7 days of indirect culture seems less harsh than for the fresh ones. While the morphology of TC-stored and PF-stored after 7 days of culture is comparable to the fresh materials, BI-stored induces no cell detachment but signs of cell apoptosis (indicated with red arrow). The storage of SG did not improve cell morphology and attachment. Just as for the fresh material, the majority of the cells are detached and clumped. It can be concluded that cell morphology and attachment are depending on material type and storage condition of 3D printed acryl-based resins.

**Figure 3:**
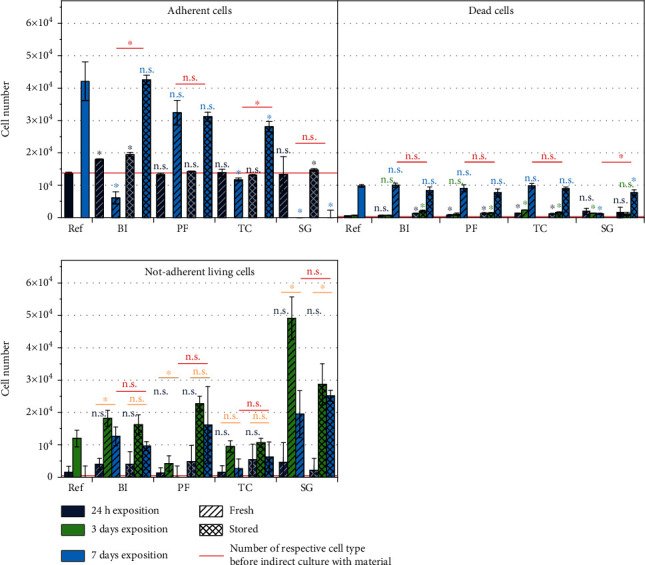
Results of spectrophotometric analyses for adherent, dead, and nonadherent living cells. Significant differences of each material compared to reference are indicated in the respective color, significant differences compared between 24 h and 7 days of indirect cell culture of each material are indicated in orange, and significant differences after 7 days culture between “fresh” and “stored” are indicated in red. Data plotted as means (*n* = 6) with SD as error bars. Significance levels *p* < 0.05 are indicated with “^∗^.” A number of adherent, dead, and nonadherent cells after 3-day expansion on the plate and before indirect culture with materials are indicated as red line, respectively.

The amount of adherent, dead, and nonadherent living cells has been determined by spectrophotometric analyses as described in the methodology (Figure 3). Adherent cells were not influenced during the first 24 h of indirect culture for the majority of materials. Interestingly, the number of living cells increased drastically after BI was treated in the storage conditions (33% for BI-fresh, 44% for BI-stored), which indicates a strong cell proliferation and will be discussed later on. The cell count development over the whole 7 days of cultivation is strongly connected to the storage of the resins. While the fresh materials mostly induced a reduction of adherent cells, stored materials allowed cells to increase in number. The improved cell tolerance after the storage is in agreement with the results of the curing level and the findings in the literature [5, 20]. BI-stored, PF-fresh, and PF-stored even reached a cell number with no significant differences to the reference after 7 days, which is an essential indicator for cytocompatible interaction. The exception to this trend is SG, where no adherent cells were determined, independently of the storage condition, supporting the qualitative results of microscopic images. It is also noteworthy that Surgical Guide is the only material that has no adherent cells after 7 days at all but still is the only material with class I certification.

Interestingly, the reason for the decreasing cell numbers is not the apoptosis of cells, as visible in the respective plot (Figure 3, dead cells). This is independent of the storage condition, although compared to the reference more cells died after 24 h and 3 days of indirect cultivation to the majority of materials. After 7 days, there are almost no significant differences between dead cells after indirect culture to the various materials compared to dead cells on the reference. Especially noteworthy are the low numbers of dead cells for SG, even though this material showed the highest impact on the adherence of cells. These results indicate a discrepancy to the number of adherent cells. In fact, the low proportion of dead cells invalidates apoptosis as a driving indicator for the cytotoxicity of the materials. Thus, the main influence of the materials is not to be attributed to the ratio of the dead cells to the total number of cells, as normally done in cytocompatibility tests. Given that hMSCs are adherent cells, meaning, they grow in a monolayer attached to the surface, reduction of adherence is a considerable change in the natural physiology of the cells. Therefore, the influence on adherence of living cells was selected as the key parameter of cell/material interaction. This is now discussed based on the number of nonadherent living cells.

In the last plot of the panel in Figure 3, the number of nonadherent living cells after 24 h, 3 d, and 7 d is depicted. After 24 hours of indirect cell culture, none of the materials and conditions show a significant difference to the reference (significance graphically shown in dark blue). After 3 days of cultivation with all materials in both storage conditions, a comparatively large proportion of nonadherent living cells becomes apparent. Since this effect is also observed in the reference, no conclusions can be drawn about material compatibility. According to the microscopic images, a higher number of nonadherent living cells were expected with longer exposure times of 7 days (significances between 24 h and 7 d graphically shown in orange). However, this was only observed as a trend without significance for BI-stored, PF-stored, TC-fresh, and TC-stored, whereas a significantly higher number of nonadherent living cells after 7 days compared to 24 h were present for BI-fresh, SG-fresh, and SG-stored. Since no consistent results were observed, a cytocompatible duration of indirect culture cannot be clearly determined.

Comparing the number of nonadherent living cells as well as the dead cells to the number of adherent cells, conclusions can be drawn about cell proliferation. For instance, during indirect culture with PF-stored, the nonadherent living cells increased compared to PF-fresh, even though the amount of adherent and dead cells is the same (Figure 3, significances graphically shown in red). Also, for BI and TC, the numbers of adherent cells differ significantly depending on the storage conditions, while no significant differences are shown neither in the dead nor in the nonadherent living cell numbers. Therefore, the influence of the material storage conditions on the number of adherent cells can only be attributed to altered proliferation. It is particularly remarkable that after indirect cultivation to BI, a significantly higher proportion of adherent cells is already observed after 24 hours. Furthermore, the same number of adherent cells was observed after 7 days of indirect cultivation to BI-stored as for the reference, although more cells died and lost adherence. Since this supportive effect on cell proliferation of a stored acrylic resin was only observed for one out of four materials, no general conclusions can be drawn about supporting proliferation capacities by stored materials. Nevertheless, a considerable impairment of the proliferation capacity of hMSC was observed after exposure to freshly printed acrylic resins (BI-fresh, PF-fresh, and TC-fresh). Storage of the materials counterbalances the negative influence of the freshly printed materials. Since only specific materials allowed adherence of cells and their proliferation on a comparable level to the reference, a resin-dependent effectiveness of storage has to be concluded.

The influence of acrylic resins on cell proliferation was also found by other groups. For instance, Nejedlá et al. found a negative effect of the class II EnvisionTEC E-Shell 300 on the proliferation of B14 cells (approx. 16%) upon direct exposure. It was demonstrated that by cleaning the resin (stirring in distilled water or 96% ethanol bath for 2× 24 hours), a comparable proliferation to the reference could be restored [15]. On the other hand, Siller et al. investigated a high-resolution polyacrylic 3D printing material for use in cultivation systems with indirect and direct contact to cells, respectively. It was stated that the investigated material induced no significant differences in cell behaviour and response, morphology, or proliferation [22]. Thus, from our present research, class I or class II certificated resins are not necessarily cytocompatible, while other acrylic resins, as proven by others, might be. Nevertheless, the cytotoxic resin residuals can be partially washed out of the bulk material with a thoroughly executed storage and posttreatment strategy. Given that all materials and conditions are impairing cell adhesion, proliferation, or at least changing cell morphology, they must be considered as limited cytocompatible.

**Figure 4:**
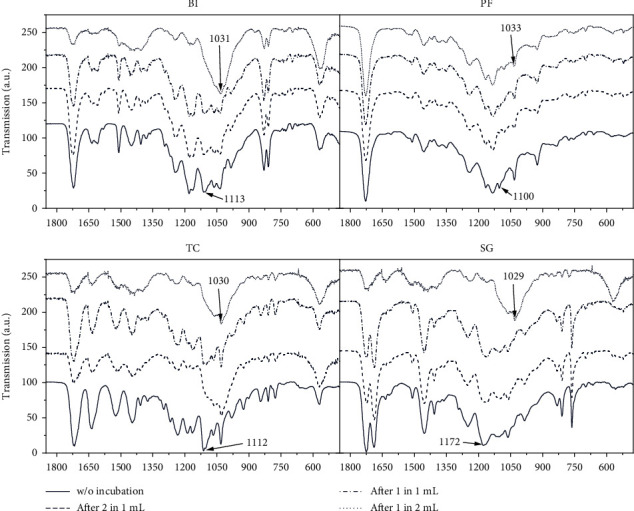
FT-IR spectrum of fresh material (cylindrical specimen with 10 mm diameter and 10 mm height) before and after incubation of 2 samples in 1 mL DMEM (“after 2 in 1 mL”), 1 sample in 1 mL (“after 1 in 1 mL”), and 1 sample in 2 mL (“after 1 sample in 2 mL”). Average spectrums of triplicates nominated from 0 to 100 are depicted.

Consequently, the influence of incubation of material specimen in cell culture medium was investigated. Therefore, a release test was performed by incubating larger cylindrical resin specimen in medium, to increase the percentage of resin residuals relative to the medium volume. This was intended to overcome potential detection limits. The specimens were incubated in descending ratios to the medium, as described in Methods. The influence of resin/liquid ratio was intended to reveal a wash-out effect of residuals as a function of the chemical structure of the resin bulk material after incubation in FT-IR. Based on the cell culture finding, this impact should be more pronounced for the fresh material, which is why only those are depicted (Figure 4).

Since the general structure of the peak position was not changed, it can be assumed that the polymer structure does not change with regard to backbone and skeleton bonds. However, the decrease of the vibrations at approx. 1030 cm^−1^ and the increase of the vibrations at approx. 1010-1110 cm^−1^ with higher medium content are noticeable. Both types of vibrations are associated with C-C stretching vibrations [23, 24]. Therefore, it can be assumed that the binding of individual side groups changes as a result of incubation. In conclusion, the side groups are affected by incubation of the freshly printed resins in the medium. Since those changes in the spectrum are particularly evident with lower resin/medium ratios, we consider the hypothesis that preliminary incubation of resin-based constructs serves as washing to increase cell/material compatibility. Furthermore, the different influences and varying degrees of cytocompatible properties of the materials on hMSCs can be assumed to be based on different polymer structures and resin formulation as seen from FT-IR results.

## 4. Conclusions

It was successfully shown that the storage time of the crosslinked acrylic resins has a significant influence on the adhesion and proliferation of the cells. In accordance with the literature, it can be stated that resin residuals diffuse out of freshly printed constructs after incubation in liquids. Those residues are most likely responsible for reduced cell activity in case of freshly printed specimen, while storage at ambient conditions increases cytocompatibility. Class I biocompatibility does not necessarily equate to good cell viability and compatibility. The Surgical Guide™ (SG) resin tested here showed by far the least cell survival owing to substantial reduction of cell adhesion. This is why no adherent cells after 7 days, regardless of storage condition, were present. Out of the selected materials and owing to the high number of living cells after 7 days compared to the reference, “Basic Ivory™” (BI) showed the highest potential for the production of in-house manufactured components for use in cell culture after a thorough cleaning and storage period. This can be stated, as long as there is no direct contact of the cells to the printed material and no attachment of the cells is needed. This result of the present work contributes to a better understanding and future applicability of the DLP resins and additive manufactured constructs.

## Data Availability

The data that support the findings of this study are available from the corresponding author upon reasonable request.

## Supplementary Materials

Supplementary Materials Table 1 with recommended printing parameters for each resin used, given by DLP printer company Zortrax (PDF).
