# Supplementary material for: Class I Biocompatible DLP-Printed Acrylate Impairs Adhesion and Proliferation of Human Mesenchymal Stromal Cells in Indirect Cytotoxicity Assay
Source: Biomed Res Int. 2023 Oct 14;2023:8305995. doi: 10.1155/2023/8305995 (PMC10590261; doi:10.1155/2023/8305995)
Supplement: Supplementary Materials — Table 1 with recommended printing parameters for each resin used, given by DLP printer company Zortrax (PDF). [file 8305995.f1.docx]

Supplementary table

Table 1: Recommended printing parameters for each resin used, given by DLP-printer company Zortrax

|  | TC | PF | BI | SG |
| --- | --- | --- | --- | --- |
| Layer | 0.025 mm | 0.025 mm | 0.025 mm | 0.025 mm |
| Layer exposure time | 10.5 s | 10.5 s | 6 s | 4 s |
| Exposure off time | 5 s | 5 s | 5 s | 5 s |
| Bottom layers exposure time | 80 s | 80 s | 60 s | 45 s |
| Bottom layers | 5 pcs | 3 pcs | 3 pcs | 5 pcs |
| Platform lower speed | 300 mm/m | 100 mm/m | 100 mm/m | 100 mm/m |
| Platform lift speed | 60 mm/m | 100 mm/m | 100 mm/m | 80 mm/m |
